# Reimagining inclusive mathematics education in Ghana through ethnomathematics

**DOI:** 10.3389/fpsyg.2026.1835750

**Published:** 2026-09-03

**Authors:** Bright Asare

**Affiliations:** Department of Mathematics Education, Akenten Appiah-Menka University of Skills Training and Entrepreneurial Development, Kumasi, Ghana

**Keywords:** curriculum, ethnomathematics, Ghana, inclusivity, mathematics

## Abstract

This study investigates the integration of ethnomathematics into Ghana's mathematics curriculum as a pathway to achieving inclusive and culturally responsive education. Despite multiple post-independence curriculum reforms, mathematics education in Ghana remains predominantly influenced by Western paradigms, often neglecting the rich indigenous mathematical knowledge embedded in the country's diverse cultural practices. This disconnects between formal schooling and the lived experiences of learners, particularly in rural and marginalized communities, contributes to low engagement, limited comprehension, and reduced academic confidence. Grounded in theories of Culturally Responsive Pedagogy, Social Constructivism, and Funds of Knowledge, the study critically examines the extent to which the current curriculum, teacher practices, and instructional resources reflect local knowledge systems. Drawing on literature, policy documents, and curriculum analysis, the paper argues the need for policy reforms, teacher training, and the systematic inclusion of ethnomathematics to foster equity, relevance, and engagement in mathematics classrooms. Ultimately, the study advocates for a culturally affirming and inclusive curriculum that bridges the gap between school mathematics and Ghana's sociocultural realities, aligning with the goals of Sustainable Development Goal 4 and Ghana's Education Strategic Plan 2018–2030.

## Introduction

Mathematics education in Ghana has historically been influenced by colonial legacies that shaped both the content and pedagogy of school curricula. Since independence, Ghana has made several efforts to reform its education system, including mathematics curricula, to meet the needs of national development and global competitiveness (Buabeng and Amo-Darko, 2026). However, these reforms have often maintained a Western-oriented structure, emphasizing abstract, decontextualized knowledge with limited connection to the lived experiences of Ghanaian learners. Consequently, the curriculum tends to overlook the rich cultural and indigenous mathematical knowledge embedded in Ghanaian society, such as that found in traditional architecture, art, trade, and games (Madu, 2025). While the introduction of standards-based curricula by the National Council for Curriculum and Assessment (NaCCA) aimed to improve critical thinking and problem-solving skills, the gap between formal school mathematics and local knowledge systems remains wide (National Council for Curriculum and Assessment, 2020).

One of the pressing challenges in Ghana's mathematics education is the lack of inclusivity in both content and pedagogical approaches (Amoako Atta and Bonyah, 2023). The dominant curriculum structure often fails to reflect the cultural identities, languages, and practices of diverse ethnic groups across the country. This disconnect contributes to alienation among students, particularly those in rural and marginalized communities, who may not see their realities represented in the classroom. Furthermore, the English-only instruction policy exacerbates comprehension difficulties and weakens learners' confidence, particularly in a multilingual society like Ghana (Nyamekye and Baffour-Koduah, 2021). As inclusivity becomes a central focus in global education agendas such as SDG 4, the need to design curricula that affirm students' cultural backgrounds and support diverse learning pathways becomes more urgent.

Integrating ethnomathematics into the Ghanaian mathematics curriculum offers a compelling solution to these challenges. Ethnomathematics, as conceptualized by Owusu-Darko et al. (2023), refers to the study of mathematical practices embedded in the cultural activities of different social groups. Applying this framework in Ghana can help learners relate mathematical concepts to familiar cultural artifacts and practices, such as kente weaving, basketry, and market calculations. This approach not only enhances conceptual understanding but also fosters cultural pride, motivation, and classroom engagement. Studies have shown that culturally responsive teaching strategies, when aligned with students' backgrounds, can lead to improved academic performance and equity in mathematics education (Nolan and Xenofontos, 2023). Therefore, embedding ethnomathematics into the national curriculum aligns with both cognitive and socio-cultural goals of education.

This paper explores the intersection of ethnomathematics and inclusive education in Ghana by examining the cultural gaps within the current curriculum, highlighting local mathematical knowledge, and advocating for policy and pedagogical reform. It situates ethnomathematics not merely as an add-on, but as a foundational principle for curriculum inclusiveness, capable of bridging the divide between formal school mathematics and Ghana's rich cultural heritage. Drawing on recent literature, curriculum policy documents, and theoretical perspectives on culturally responsive pedagogy, the study contributes to the discourse on transforming mathematics education to be more equitable, context-relevant, and empowering for all learners in Ghana.

## Conceptual framework and theoretical underpinnings

### Ethnomathematics defined

Ethnomathematics, a term first coined by Brazilian mathematician D'Ambrosio (2013), refers to the study of mathematical practices embedded within distinct cultural traditions. According to D'Ambrosio (2019), ethnomathematics is “a program in the history and philosophy of mathematics with an educational perspective, based on the recognition that different cultural groups develop their ways of doing mathematics,” as cited by Turmuzi et al. (2023). Scholars such as Sata (2024) have expanded on this definition by emphasizing ethnomathematics as a tool for educational equity, arguing that it repositions indigenous mathematical knowledge within formal schooling. Key components of ethnomathematics include culture-based practices, local systems of measurement, spatial reasoning in architecture, and counting systems used in everyday social practices like trade and farming (Marbun et al., 2026). These practices offer contextual and experiential learning opportunities that reflect students' lived realities, making mathematics more meaningful and accessible. Despite structural differences between school mathematics, which is often abstract and Eurocentric, and indigenous mathematical systems, which are practical and contextual, both forms seek to explain patterns, quantities, and spatial relationships, revealing complementary perspectives rather than oppositional ones.

### Inclusive mathematics curriculum

An inclusive mathematics curriculum ensures equitable access to learning opportunities by acknowledging and incorporating the diverse needs, experiences, and cultural identities of all learners. According to Kaur et al. (2017), indicators of inclusiveness include curriculum content that is linguistically and culturally responsive, instructional practices that accommodate learner variability, and assessments that are fair and representative. The goals of an inclusive curriculum encompass accessibility, equity, relevance, and participation, especially for marginalized and linguistically diverse learners (Bakogiannis and Papavasiliou, 2026). Within the context of Ghana, where students come from various ethnic and linguistic backgrounds, an inclusive curriculum requires the deliberate integration of indigenous knowledge and real-life experiences into the teaching of mathematics (Boakye-Yiadom et al., 2025).

The universal Design for Learning (UDL) framework supports this approach by promoting multiple means of representation, engagement, and expression, thereby addressing the variability in students' learning profiles (Kelly et al., 2022). By embedding ethnomathematical elements, such as local symbols or measurement systems, into mathematics instruction, the curriculum becomes more culturally affirming and pedagogically inclusive.

**Figure 1:**
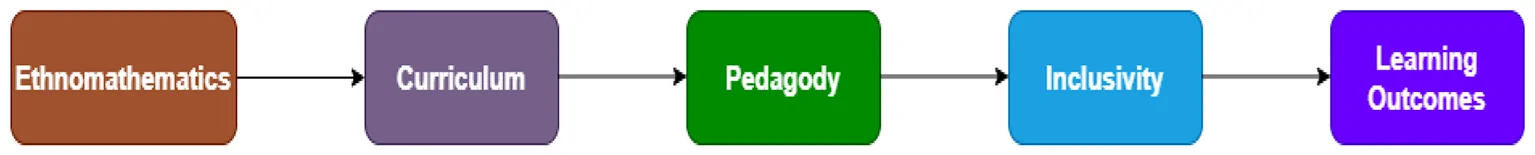
Conceptual framework (Source: Author Creation, 2026).

Figure 1 presents the conceptual framework underpinning this study. It illustrates how ethnomathematics provides the cultural foundation for designing a mathematics curriculum that reflects learners' everyday experiences, indigenous knowledge, and local practices in Ghana. When these cultural perspectives are embedded in the curriculum, they influence the choice of teaching methods, encouraging teachers to adopt learner-centered and culturally responsive pedagogies. Such pedagogical approaches create more inclusive classrooms by valuing learners' diverse cultural backgrounds, prior knowledge, and ways of thinking, thereby ensuring that every learner has meaningful opportunities to participate in mathematics learning. As a result, students are more likely to develop deeper conceptual understanding, greater confidence, improved problem-solving skills, and positive attitudes toward mathematics. In this way, the framework demonstrates that integrating ethnomathematics into the curriculum and pedagogy is a practical pathway to reimagining a more inclusive, equitable, and contextually relevant mathematics education in Ghana.

### Theoretical lenses

Understanding ethnomathematics and inclusive curriculum design requires a grounding in educational theories that emphasize culture, identity, and social context. Culturally Responsive Pedagogy (Gay, 2015) posits that teaching should affirm students' cultural backgrounds and experiences while promoting academic success, as cited by Gist (2017). In mathematics education, this involves contextualizing problems in students' cultural settings and incorporating indigenous knowledge to build conceptual understanding. Vygotsky's Social Constructivism (Vygotsky 2019) further informs this approach by highlighting the importance of social interaction and cultural tools in cognitive development, as cited by Daramola et al. (2024). Mathematical understanding is socially mediated, and learners co-construct knowledge through language, community practices, and culturally meaningful activities. Similarly, the Funds of Knowledge framework by Llopart and Esteban-Guitart (2018) suggests that households and communities possess valuable intellectual resources that should be harnessed in classroom instruction. Recent studies (e.g., Dooty et al., 2026) advocate leveraging students' home-based mathematical experiences, such as traditional craft design or market arithmetic, to enrich mathematics learning and foster inclusivity.

### Bridging theory and practice in Ghanaian education

Applying these theoretical lenses to the Ghanaian educational context reveals both challenges and opportunities. While curriculum policy documents, such as those developed by the National Council for Curriculum and Assessment (NaCCA), promote learner-centered and inclusive approaches, practical implementation often falls short due to a lack of teacher training and inadequate resources. Ethnomathematics offers a pathway to bridge formal school mathematics with learners' sociocultural realities, but only if teachers are equipped to identify and integrate culturally relevant content (Asare, 2026). For instance, incorporating Adinkra symbols into lessons on symmetry or using traditional architecture to teach geometry allows students to relate abstract concepts to familiar contexts. Such approaches align with UDL principles and the pedagogical ideals of Vygotsky, Gay, and Moll, emphasizing the construction of knowledge and validation of students' cultural identities. As Ghana strives toward inclusive education goals under the Education Strategic Plan 2018–2030, embedding ethnomathematics in mathematics curricula can contribute to a more culturally sustaining and equitable educational system (Ofori Nyarko, 2026).

## Ethnomathematics in the Ghanaian context

### Cultural and ethnic diversity in Ghana

Ghana is characterized by a rich tapestry of cultural and ethnic diversity, with *over 100 ethnic* groups, including the Akan, Mole-Dagbani, Ewe, Ga-Dangme, and Guan, each possessing distinct languages, traditions, and socio-cultural practices that embody mathematical knowledge. These cultural groups exhibit mathematical reasoning through daily activities such as construction, craftwork, trade, and games, which offer insights into locally embedded mathematical knowledge systems. For instance, the complex symmetrical patterns in *kente* weaving among the Akan people demonstrate principles of geometry and transformation, while traditional architectural layouts among the Ga and Dagomba often apply concepts of measurement, proportion, and spatial reasoning (Atta et al., 2025). Furthermore, farming calendars based on lunar cycles and seasonal calculations across various ethnic groups represent mathematical patterns in time measurement, probability, and estimation (Comia et al., 2026). These examples reflect the naturally occurring ethnomathematical practices embedded within the cultural heritage of Ghana.

These localized practices demonstrate rich mathematical thinking often overlooked in formal education. Indigenous games like *Oware* (a traditional counting game) support the development of arithmetic skills and logical reasoning, while market trade practices involve estimation, mental calculations, unit conversions, and bargaining techniques grounded in practical mathematics. The symbolic meanings in *Adinkra* symbols, used by the Ashanti people, integrate geometric symmetry, recursion, and binary logic, making them powerful tools for connecting abstract mathematical concepts to cultural meaning. Despite being informal, these mathematical elements are robust, complex, and pedagogically relevant, aligning well with school-based content in number sense, algebra, and pattern recognition. Ethnomathematics, therefore, presents a culturally affirming lens through which students can relate mathematical concepts to their lived experiences, enhancing both cognitive and affective engagement (Payadnya et al., 2025).

### Disconnect between mainstream curriculum and indigenous knowledge

Despite the cultural richness of Ghana's ethnomathematical landscape, the national mathematics curriculum remains predominantly Western in orientation (Age and Machaba, 2026; Asare, 2026). Influenced heavily by colonial-era British curriculum models, the present-day syllabus largely emphasizes abstract and decontextualized mathematics, with limited space for local or indigenous knowledge (Asare, 2026). This creates a disconnect between the home-based mathematical experiences of students and the formal instruction they receive in schools, contributing to disinterest, underachievement, and feelings of cultural alienation in mathematics classrooms. The curriculum framework, while emphasizing competencies and critical thinking, rarely incorporates indigenous activities like weaving, trading, or farming as valid mathematical contexts. This omission implicitly marginalizes the everyday mathematical practices of students and their communities.

Ethnomathematical practices in Ghana align strongly with key strands of the formal mathematics curriculum, particularly number, geometry, and algebra. For example, market trading activities naturally develop number concepts such as arithmetic operations, estimation, ratios, and unit conversions, while indigenous games like *oware* support logical reasoning and pattern recognition linked to early algebraic thinking. Similarly, kente weaving, basketry, and Adinkra symbol design embody geometric concepts including symmetry, transformation, tessellation, and spatial reasoning, which are explicitly emphasized in the geometry strand of the curriculum. However, there is a need for caution against romanticizing culture or oversimplifying indigenous knowledge by treating cultural practices as mere teaching aids without acknowledging their complexity and contextual meaning. When cultural examples are superficially inserted, they risk reinforcing stereotypes or distorting authentic knowledge systems. Therefore, effective curriculum integration requires deliberate alignment of ethnomathematical examples with clearly defined curriculum objectives, ensuring that indigenous practices are used analytically to deepen conceptual understanding rather than symbolically, thereby maintaining both cultural integrity and mathematical rigor.

Moreover, instructional materials such as textbooks and teacher guides rarely reference or build on local knowledge systems (Chavula et al., 2022). A recent content analysis of Ghanaian primary and junior high school mathematics textbooks revealed an overwhelming reliance on Eurocentric examples and scenarios, with fewer than 5% of tasks referencing local settings or cultural artifacts (Nyamekye and Uwen, 2024). This alienation is further exacerbated by linguistic barriers; while students often learn mathematics in English, a second or third language for many, there is minimal effort to translate or contextualize mathematical terminology in indigenous languages. Consequently, learners struggle with both conceptual and linguistic access to mathematical ideas (Maanu and Asare, 2025). The cumulative effect is a form of epistemological exclusion, where formal mathematics education systematically devalues the cognitive legitimacy of indigenous knowledge. Bridging this gap is critical to fostering inclusive, relevant, and equitable mathematics education in Ghana.

## Curriculum analysis and pedagogical gaps

### Review of Ghana's mathematics curriculum: content structure and cultural representation

The current mathematics curriculum developed by the National Council for Curriculum and Assessment (NaCCA) in Ghana aims to foster critical thinking, problem-solving, and mathematical reasoning. However, a closer analysis reveals a predominant reliance on Western mathematical constructs with minimal integration of culturally grounded examples. While the curriculum emphasizes competencies such as numeracy and spatial reasoning, it largely omits indigenous mathematical knowledge systems that exist within Ghana's diverse ethnic communities (Zoogah, 2023). Concepts such as patterns, symmetry, and estimation, which are inherently present in traditional crafts like kente weaving or Adinkra symbol design, are rarely contextualized using these local practices. This omission reflects a disconnect between the lived mathematical experiences of students and the formal school curriculum, reducing opportunities for culturally relevant learning.

### Teacher guides and assessment practices

The teacher resource packs and guides provided by NaCCA do attempt to offer pedagogical strategies aligned with competency-based education, yet they often fall short in incorporating local knowledge systems as instructional tools. For instance, the guides focus on generic teaching strategies such as group work and real-life application, but provide limited culturally relevant examples that connect with students' sociocultural backgrounds (Hayashi et al., 2022). Furthermore, the assessment practices remain largely summative and structured in standardized formats that do not consider diverse ways of demonstrating mathematical understanding rooted in local knowledge. Consequently, learners whose cognitive development is shaped by indigenous contexts may struggle to align their understanding with school-based assessments, thus affecting both performance and confidence (Hayes, 2003).

### Inclusiveness of local examples and multilingual support

One critical shortcoming of Ghana's mathematics curriculum is its limited use of local examples and absence of multilingual integration, especially in lower primary, where students are more comfortable with mother tongue instruction. Despite Ghana's language-in-education policy advocating for local language use in early grades, mathematics textbooks and materials continue to be predominantly in English, thereby restricting accessibility and comprehension (Hankerson and Obiri-Yeboah, 2026). Additionally, examples provided in textbooks rarely reflect students' cultural or environmental experiences, such as market trading, traditional architecture, or farming practices. This lack of localization and linguistic inclusiveness reinforces inequity in mathematics education, particularly in rural or linguistically diverse regions where students' cultural capital is not validated in the learning process.

### Teacher preparedness and beliefs

Teachers in Ghana face considerable challenges in implementing ethnomathematics due to limited training in culturally responsive pedagogy. Most teacher education programs focus primarily on Western-oriented mathematical theories and procedural fluency, leaving preservice and in-service teachers underprepared to integrate indigenous mathematical practices in their instruction (Gregory, 2009). Moreover, professional development workshops rarely address how to incorporate students' cultural experiences into the mathematics classroom (Johnson and Elliott, 2020). Teachers' beliefs also play a crucial role; many views traditional knowledge as informal, unscientific, or irrelevant to standardized curricula, thus hesitating to explore ethnomathematics despite its potential to improve engagement and understanding (Kumar and Gopinath, 2025). Without targeted policy and training interventions, this ideological gap risks perpetuating a curriculum that is culturally alienating to learners and inconsistent with Ghana's educational inclusivity goals.

### Conceptual review

#### Mathematics education

Mathematics education is concerned with how mathematical knowledge is taught, learned, and applied in different educational contexts. It encompasses not only the acquisition of mathematical concepts and computational skills but also the development of learners' ability to reason logically, solve problems, communicate mathematical ideas, and apply mathematics to everyday situations. Over the years, the focus of mathematics education has evolved from emphasizing memorization of rules and procedures to promoting conceptual understanding, critical thinking, and active learner engagement (Dairo et al., 2024). This shift reflects the growing recognition that learners develop a deeper understanding of mathematics when they are actively involved in constructing knowledge rather than simply receiving information from their teachers. In Ghana, mathematics is regarded as one of the core subjects at all levels of basic and secondary education because of its importance in science, technology, engineering, business, and national development. It is also viewed as an essential tool for developing analytical thinking and decision-making skills that learners need both in school and in everyday life. Despite its recognized importance, mathematics remains one of the subjects that many Ghanaian learners find difficult (Ampadu and Danso, 2018). National examination reports have consistently revealed poor performance in mathematics among learners at both the basic and secondary school levels. Researchers have attributed these challenges to several factors, including teacher-centered instructional approaches, limited instructional resources, learners' anxiety toward mathematics, language difficulties, and inadequate connections between classroom mathematics and learners' everyday experiences (Yu and Singh, 2018). These concerns have generated increasing interest in instructional approaches that make mathematics more meaningful and relevant to learners' social and cultural contexts.

#### The nature of mathematics

The way mathematics is understood influences how it is taught and learned. For many years, mathematics was regarded as a fixed body of knowledge consisting of universal laws, formulas, and procedures that learners were expected to memorize and reproduce accurately. This traditional perspective portrays mathematics as objective and independent of human culture. While this view highlights the precision and logical structure of mathematics, it overlooks the social and cultural processes through which mathematical ideas have developed over time. Contemporary scholars argue that mathematics should instead be viewed as a dynamic human endeavor that evolves in response to the needs of society. Fouze and Amit (2018) maintains that mathematical knowledge is constructed through human interaction and is influenced by historical and cultural experiences. Likewise, Papandreou and Tsiouli (2022) explains that mathematics originates from common human activities such as counting, measuring, designing, locating, playing, and explaining. Every society performs these activities, although the methods and symbols used may differ across cultures. Consequently, mathematics should not be seen as belonging to a particular civilization but as knowledge that has developed through the contributions of different cultures over many centuries. This broader understanding of mathematics is particularly important within the Ghanaian context. Long before the introduction of formal Western education, indigenous communities employed mathematical ideas in farming, trading, architecture, weaving, pottery, bead making, and various other occupations. For example, Kente weavers create complex geometric patterns based on symmetry and repetition, market traders perform rapid mental calculations during transactions, and traditional builders apply principles of measurement and proportion when constructing houses. These practices demonstrate that mathematics exists naturally within everyday cultural activities. Recognizing such indigenous mathematical knowledge broadens learners' understanding of mathematics and helps them appreciate its relevance beyond the classroom.

#### Mathematics as a human activity

The view of mathematics as a human activity has become increasingly influential in mathematics education. Tachie (2019) argued that mathematics is not simply a body of knowledge to be transmitted from teacher to learner; rather, it is an activity through which individuals organize and make sense of the world around them. According to this perspective, learners should experience mathematics by exploring, investigating, discussing, and solving meaningful problems instead of memorizing isolated procedures. Human societies have always developed mathematical ideas in response to practical needs. Communities have created methods of counting livestock, measuring farmland, constructing buildings, organizing trade, and predicting seasonal changes. As these activities evolved, so too did mathematical knowledge. This demonstrates that mathematics is closely connected to the cultural, economic, and environmental circumstances in which people live. The concept of ethnomathematics reinforces this perspective. Clivaz and Miyakawa (2020) argues that every cultural group develops mathematical knowledge through its daily activities and problem-solving practices. In Ghana, examples of such knowledge are abundant. Farmers estimate planting distances and calculate expected yields, traders determine profits and discounts mentally, artisans create symmetrical patterns in textiles and pottery, while carpenters and builders use measurement and geometry in construction. These examples illustrate that mathematical thinking is embedded within ordinary human activities and is not restricted to formal schooling. Viewing mathematics as a human activity has important implications for teaching. It suggests that teachers should build on learners' everyday experiences and cultural knowledge when introducing mathematical concepts. Doing so enables learners to recognize mathematics as something they already encounter in their communities rather than as an unfamiliar and abstract subject.

#### Mathematics as problem solving

Problem solving is widely regarded as one of the primary goals of mathematics education. Mathematics is not simply about obtaining correct answers; it is about using logical thinking to understand situations, identify relationships, and develop appropriate solutions. Learners are expected to analyze problems, select suitable strategies, justify their methods, and evaluate the reasonableness of their answers. Pólya (1945) described problem solving as a systematic process involving four stages: understanding the problem, devising a plan, implementing the plan, and reviewing the solution, as cited by Fernandez et al. (2019). This framework remains highly influential because it encourages learners to think critically rather than relying solely on memorized procedures. Similarly, Sengupta-Irving and Agarwal (2017) argues that successful problem solving requires more than mathematical knowledge; learners also need strategic thinking, persistence, and the ability to monitor their own reasoning throughout the solution process. In contemporary mathematics education, problem solving is viewed not only as an objective of instruction but also as a powerful method of learning mathematics. When learners solve meaningful problems, they actively construct new knowledge by connecting prior experiences with new mathematical ideas (Rehman et al., 2024). Such learning experiences promote conceptual understanding, creativity, and confidence. Within many Ghanaian classrooms, however, mathematics instruction often emphasizes routine exercises and examination preparation. Learners are frequently taught standard procedures for solving textbook questions without sufficient opportunities to explore unfamiliar problems or develop their own solution strategies. Although procedural fluency is important, overreliance on routine exercises may limit learners' ability to apply mathematics in practical situations. Integrating problems drawn from learners' everyday experiences, such as market transactions, farming, weaving, and local construction, can help learners appreciate the usefulness of mathematics while strengthening their problem-solving skills.

#### Mathematics as communication

Communication is an essential aspect of mathematical learning because it enables learners to express ideas, explain their thinking, and learn from others. Mathematics has its own language, consisting of symbols, diagrams, graphs, formulas, and technical vocabulary. However, mathematical communication extends beyond symbolic notation. It includes discussing ideas, asking questions, explaining reasoning, interpreting representations, and engaging in collaborative problem solving. According to the National Council of Teachers of Mathematics (2000), learners develop deeper mathematical understanding when they are given opportunities to communicate their ideas clearly and to evaluate the reasoning of others, as cited by Webb et al. (2023). Through classroom discussions, learners clarify misconceptions, strengthen conceptual understanding, and develop confidence in expressing mathematical ideas. Vygotsky's (2019) sociocultural theory further emphasizes that learning takes place through interaction with others. Classroom dialogue allows learners to negotiate meaning, receive feedback, and gradually internalize mathematical concepts. Teachers therefore play an important role in creating learning environments where learners feel comfortable discussing mathematical ideas and exploring different solution strategies. In Ghana, effective mathematical communication is influenced by language. Since English serves as the official language of instruction while many learners speak indigenous languages at home, language barriers sometimes hinder conceptual understanding. Young learners, in particular, often find it easier to grasp mathematical concepts when teachers explain ideas using familiar local languages alongside English. Ethnomathematics provides additional opportunities for meaningful communication by encouraging learners to discuss mathematical ideas through familiar cultural examples and indigenous knowledge.

#### Mathematics as reasoning

Reasoning is central to mathematical thinking because it enables learners to explain why mathematical ideas are true rather than simply accepting them as facts. Mathematical reasoning involves identifying patterns, making logical connections, drawing conclusions, constructing arguments, and justifying solutions. These processes help learners develop a deeper understanding of mathematics and become independent thinkers. Different forms of reasoning are used in mathematics, including inductive reasoning, deductive reasoning, proportional reasoning, algebraic reasoning, and statistical reasoning (Chimoni and Pitta-Pantazi, 2017). Inductive reasoning involves generalizing from observed patterns, whereas deductive reasoning applies established principles to reach logically valid conclusions. Both forms contribute to the development of mathematical proficiency (Choi et al., 2026). Reasoning is not confined to formal school mathematics. Everyday activities within Ghanaian communities also require sophisticated mathematical reasoning. Farmers estimate crop yields based on previous harvests, traders compare prices and calculate profits mentally, weavers organize complex geometric patterns, and builders determine appropriate measurements for construction. These examples demonstrate that learners often possess valuable reasoning skills before entering the classroom. Recognizing these informal forms of reasoning provides opportunities for teachers to connect school mathematics with learners' lived experiences.

#### Current teaching methods in Ghana

Mathematics teaching in Ghana has changed considerably in recent years following curriculum reforms aimed at improving the quality of education. The Standards-Based Curriculum at the primary level and the Common Core Program at the junior high school level emphasize learner-centered teaching approaches that promote critical thinking, collaboration, communication, creativity, and problem solving (National Council for Curriculum and Assessment, 2019). These reforms encourage teachers to facilitate learning rather than simply transmit knowledge. Despite these policy changes, classroom practice often continues to reflect traditional approaches. In many schools, mathematics lessons remain dominated by teacher explanations, demonstrations of procedures, note-taking, and repetitive practice exercises designed to prepare learners for examinations (Awaah et al., 2023; Kusi et al., 2026). Although these methods help learners acquire procedural skills, they frequently provide limited opportunities for learners to investigate mathematical ideas independently or relate mathematics to their own experiences. Many teachers also use question-and-answer techniques during lessons, but these questions often require short factual responses instead of encouraging learners to explain their reasoning or consider multiple approaches to solving problems. Group work and activity-based learning are becoming more common, particularly in schools implementing the competency-based curriculum. However, their effectiveness is often constrained by large class sizes, inadequate teaching resources, limited professional development opportunities, and pressure to complete the prescribed syllabus. At the primary level, teachers commonly use concrete teaching materials such as counters, bottle tops, sticks, beads, and locally available objects to introduce mathematical concepts. While these materials help learners develop conceptual understanding, instruction frequently shifts too quickly toward abstract symbolic representations. As a result, some learners struggle to make meaningful connections between concrete experiences and formal mathematical ideas. Another important concern is that classroom mathematics often reflects examples drawn from textbooks rather than learners' cultural environments. Consequently, many learners perceive mathematics as abstract and unrelated to their daily lives. Integrating ethnomathematical approaches into classroom instruction offers an opportunity to address this gap. Drawing on local cultural practices such as market trading, farming, traditional architecture, weaving, bead making, and indigenous games can make mathematics more relevant, meaningful, and inclusive for Ghanaian learners. Such an approach is particularly important in the context of inclusive education because it recognizes learners' cultural backgrounds as valuable resources for learning. By connecting school mathematics with indigenous knowledge and everyday experiences, teachers can enhance learner engagement, improve conceptual understanding, and promote equitable participation in mathematics classrooms.

## Methodology

### Search strategy

This study employed a systematic narrative review to explore the role of ethnomathematics in promoting inclusive mathematics education in Ghana. A structured search strategy was used to identify relevant literature by combining keywords such as *ethnomathematics, inclusive mathematics education, culturally responsive teaching, indigenous knowledge*, and *Ghana mathematics curriculum* using Boolean operators (AND, OR). The search was designed to capture studies that examined the relationship between culture, mathematics learning, and curriculum development, while ensuring that the review remained focused on the objectives of the study.

### Databases consulted

Relevant studies were obtained from several reputable academic databases, including Scopus, Web of Science, ERIC, Google Scholar, ScienceDirect, SpringerLink, Taylor and Francis Online, and JSTOR. In addition, official policy and curriculum documents were retrieved from the websites of the National Council for Curriculum and Assessment (NaCCA), the Ghana Education Service (GES), the Ministry of Education, and UNESCO. Using both scholarly publications and policy documents provided a comprehensive understanding of the current state of inclusive mathematics education and curriculum development in Ghana.

### Inclusion and exclusion criteria

The review included peer-reviewed journal articles, books, book chapters, and official policy documents that focused on ethnomathematics, inclusive mathematics education, culturally responsive teaching, indigenous knowledge systems, or mathematics curriculum development. Only studies published in English and directly related to the Research Topic were considered. Studies that did not address mathematics education, lacked sufficient academic evidence, were duplicate publications, or were unavailable in full text were excluded from the analysis to maintain the quality and relevance of the review.

**Figure 2:**
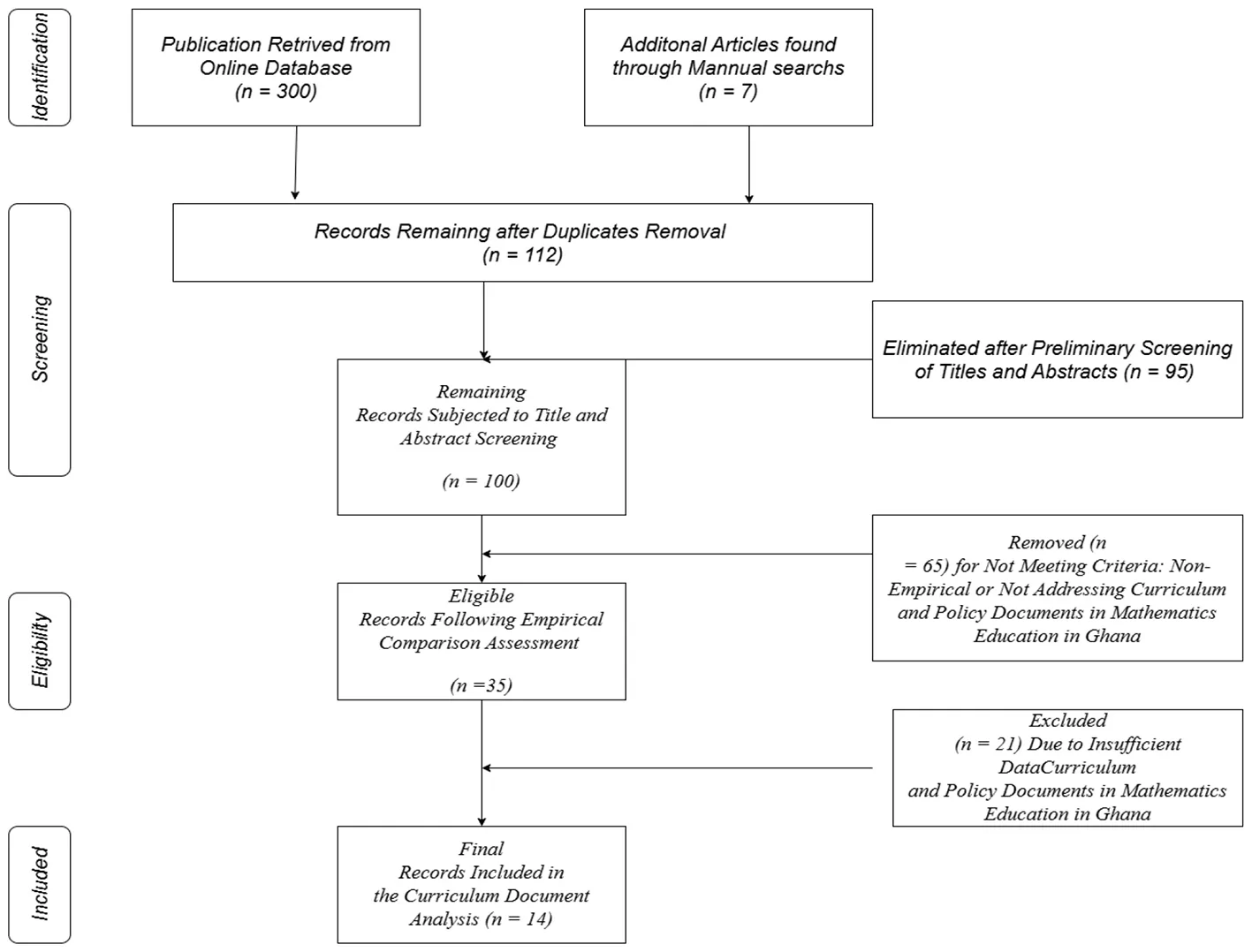
Flowchart of the study screening and selection procedure (Source: Author, Design, 2026).

Figure 2 illustrates the process used to identify and select curriculum and policy documents for this review. The search initially identified 307 publications, consisting of 300 records retrieved from online databases and 7 additional records obtained through manual searches. After duplicate records were removed, 112 publications remained. From these, 100 records were screened based on their titles and abstracts, leading to the exclusion of 95 studies that did not meet the initial inclusion criteria. The remaining records were then assessed in greater detail to determine their relevance and methodological suitability. Studies that were non-empirical or did not focus on curriculum and policy documents in mathematics education in Ghana were excluded, while others were removed because they contained insufficient information to address the objectives of the review. Following this rigorous screening and eligibility assessment, 14 curriculum and policy documents met all the inclusion criteria and were retained for the final analysis. This systematic selection process ensured that the review was based on the most relevant and credible evidence available, thereby strengthening the reliability and validity of the study's findings.

### Period covered

The review focused on literature published from 2017 and 2026, a period that reflects major developments in inclusive education, curriculum reform, and culturally responsive teaching both globally and within Ghana. This timeframe also captures the transition toward standards-based curriculum reforms introduced in Ghana and the growing recognition of indigenous knowledge as a valuable resource for teaching and learning mathematics.

### Document selection process

The document selection followed a systematic process. First, all retrieved records were screened to remove duplicate entries. The titles and abstracts of the remaining studies were then reviewed to determine their relevance to the research objectives. Articles that appeared suitable were read in full before making the final selection. Curriculum and policy documents were purposively selected based on their relevance to mathematics education in Ghana. Only documents that provided meaningful evidence for understanding the integration of ethnomathematics into inclusive mathematics education were included in the final review. Table 1 summary of research articles related to curriculum and policy documents in mathematics education in Ghana.

**Table 1:** Summary of research articles related to curriculum and policy documents in mathematics education in Ghana.

No	Article/research work	Author(s)	Year	Relevance to mathematics education in Ghana
1	Global proficiency framework: Analysis of national and Colleges of Education curricula in Ghana	Amusuglo and Jančarík	2024	Examines how Ghana's early-grade mathematics curriculum and teacher education curriculum align with international mathematics proficiency standards. Useful for curriculum policy analysis.
2	High school teachers' perceptions and practices of mathematics curriculum in Ghana	Oppong-Gyebi et al.	2023	Investigates SHS mathematics teachers' views about curriculum relevance, implementation challenges, and classroom practices.
3	Analyses of algebra knowledge and difficulties among Ghanaian Junior High School learners: insights from standard-based mathematics curriculum implementation	Osei and Agyei	2024	Studies learners' algebra understanding under Ghana's new standards-based mathematics curriculum.
4	Review of literature on teaching and learning geometry and measurement: a case of Ghanaian standards-based mathematics curriculum	Baah-Duodu et al.	2020	Reviews the development of geometry and measurement strands in Ghana's standards-based curriculum and informs curriculum review processes.
5	Teachers' experiences implementing Ghana's common core mathematics curriculum through the lens of the concerns-based adoption model	Manfred et al.	2026	Examines teachers' experiences implementing Ghana's Common Core Program mathematics curriculum and policy reforms.
6	Exploring valuing pedagogies across grade levels: insights from Ghana's standards-based mathematics curricula	Nyarko and Chiu	2026	Analyses how values and competencies are embedded in Ghanaian mathematics curriculum documents.
7	Values and valuing pedagogies in the Ghanaian mathematics curricula	Ofori Nyarko	2026	Uses curriculum content analysis to examine how values are represented in Ghana's mathematics curriculum.
8	A critical review of Ghana's primary mathematics curriculum (B1–B6): structural analysis, challenges, and directions for reform	Mireku	2020	Critically evaluates the structure, standards, indicators, and challenges of Ghana's primary mathematics curriculum.
9	A policy perspective on integrating STEM education into Ghana's foundational curriculum	Buabeng and Amo-Darko	2026	Discusses curriculum policy reforms and STEM integration within Ghana's foundational education system, including mathematics.
10	The development and implementation of Ghana's standards-based curriculum: implications for mathematics teaching and learning	(Curriculum reform researchers)	2020–2022	Relevant for understanding the shift from content-based teaching to competency-based mathematics education.
11	National curriculum framework for pre-tertiary education in Ghana: issues and implications for mathematics education	Ministry of Education/NaCCA-related studies	2018 onwards	Examines the policy foundation behind competency-based mathematics curriculum reforms.
12	National Teacher Education Curriculum Framework (NTECF) and mathematics teacher preparation in Ghana	Ghana Ministry of Education researchers	2017 onwards	Important for understanding how teacher preparation policies influence mathematics curriculum implementation.
13	Curriculum reform and mathematics achievement in Ghanaian basic schools	Ghanaian education researchers	Various years	Links curriculum reforms with mathematics achievement, assessment outcomes, and learner performance.
14	Assessment policy and mathematics learning outcomes in Ghanaian schools	Ghana education assessment researchers	Various years	Examines the relationship between national assessment policies (BECE/WASSCE) and mathematics teaching practices.

### Criteria for curriculum document analysis

The curriculum documents were examined to determine how they address inclusion, cultural diversity, and the integration of indigenous mathematical knowledge into classroom practice. Particular attention was given to curriculum goals, learning outcomes, suggested teaching approaches, assessment practices, and opportunities for incorporating learners' cultural experiences into mathematics instruction. The analysis also explored whether the curriculum encourages equitable participation and supports teaching strategies that recognize Ghana's diverse cultural and linguistic backgrounds, thereby promoting a more inclusive mathematics learning environment. Table 2 analysis of Ghana's curriculum and policy documents on curriculum goals, learning outcomes, teaching approaches, assessment practices, inclusion, cultural diversity, and indigenous mathematical knowledge in mathematics education.

**Table 2:** Analysis of Ghana's curriculum and policy documents on curriculum goals, learning outcomes, teaching approaches, assessment practices, inclusion, cultural diversity, and indigenous mathematical knowledge in mathematics education.

Curriculum/policy document	Curriculum goals	Learning outcomes	Teaching approaches	Assessment practices	Inclusion, Cultural diversity, and indigenous mathematical knowledge
National Pre-Tertiary Education Curriculum Framework (Ministry of Education (MoE), 2018)	Develop critical thinking, creativity, problem-solving, collaboration, and lifelong learning.	Learners demonstrate mathematical reasoning, communication, and application to real-life situations.	Learner-centered, inquiry-based, collaborative learning, problem-solving, and differentiated instruction.	Continuous assessment emphasizing knowledge, skills, attitudes, and values.	Promotes inclusive education by recognizing learner diversity, encouraging equal participation, and using local examples that reflect Ghanaian cultural experiences.
Standards-Based Curriculum (KG–Basic 6) (National Council for Curriculum and Assessment, 2019)	Develop mathematically literate citizens capable of solving everyday problems.	Learners apply mathematics in familiar and unfamiliar contexts using conceptual understanding.	Activity-based learning, hands-on investigations, games, cooperative learning, manipulatives, and contextual learning.	Formative and summative assessments aligned with performance standards.	Encourages teachers to use local materials, cultural artifacts, market activities, traditional games, and community practices to teach mathematical concepts. Supports learners with different abilities through differentiated instruction.
Common Core Programme (Basic 7–10) (National Council for Curriculum and Assessment, 2020)	Develop competencies in critical thinking, communication, digital literacy, and innovation.	Learners solve authentic mathematical problems and make informed decisions using mathematics.	Project-based learning, collaborative problem-solving, ICT integration, inquiry, and real-life applications.	Competency-based assessment using projects, portfolios, classroom activities, and written tests.	Promotes culturally responsive teaching by connecting mathematics to learners' communities, occupations, local industries, and indigenous practices while ensuring gender equity and inclusion.
National Teacher Education Curriculum Framework (NTECF) (Ministry of Education (MoE), 2017)	Prepare competent, reflective, and inclusive teachers.	Student teachers develop pedagogical content knowledge and inclusive teaching competencies.	Reflective practice, lesson study, collaborative learning, classroom research, and culturally responsive pedagogy.	School-based assessment, teaching practice, portfolios, and reflective journals.	Emphasizes inclusive pedagogy, multicultural education, gender sensitivity, Universal Design for Learning (UDL), and adapting mathematics instruction to learners' cultural backgrounds.
Education Strategic Plan (ESP)	Improve quality, equity, access, and relevance of education.	Improve learning achievement and reduce disparities among learners.	Supports teacher professional development and equitable resource distribution.	National monitoring and learning achievement assessments.	Focuses on equitable access for girls, children with disabilities, rural learners, and marginalized communities while promoting culturally relevant education.
National Education Reform Program	Transform education to meet national development needs through competency-based learning.	Equip learners with employability, innovation, and practical problem-solving skills.	Competency-based instruction, interdisciplinary learning, digital learning, and experiential activities.	Continuous assessment integrated with national examinations.	Encourages contextualized learning using local knowledge, community resources, and learners' experiences to strengthen mathematical understanding.

## Discussion

Despite the strong theoretical and pedagogical case for integrating ethnomathematics into Ghana's mathematics curriculum, several implementation challenges hinder its widespread adoption. One critical barrier is teacher workload and preparedness; as noted in the manuscript, many teachers receive training that emphasizes Western-oriented mathematical procedures, leaving them underprepared to identify, contextualize, and integrate indigenous mathematical practices such as kente weaving patterns, Adinkra symbols, or market arithmetic into lessons. This gap is compounded by a lack of institutional support, as professional development programs rarely provide practical guidance or follow-up coaching on ethnomathematics. Exam alignment also presents a challenge: standardized assessments and summative evaluations remain largely Eurocentric and abstract, failing to recognize culturally embedded problem-solving approaches. Consequently, teachers may resist incorporating ethnomathematical content, fearing that students' performance in national exams could be negatively affected (Sunzuma and Maharaj, 2019). Additionally, resource constraints, including the scarcity of culturally relevant textbooks, instructional materials, and visual aids, further limit the feasibility of implementation, particularly in rural schools where local examples might differ across communities. Examples from Ghana indicate that attempts to embed ethnomathematics in classrooms have occasionally faced resistance; for instance, teachers often regard indigenous knowledge as informal or irrelevant to formal mathematics learning, leading to minimal integration despite policy encouragement. These challenges highlight the need for systemic reforms, including targeted teacher training, development of culturally responsive assessment tools, and provision of local instructional resources, to ensure that ethnomathematics can be meaningfully incorporated without overburdening educators or compromising examination outcomes.

## Conclusions

This study makes three significant contributions to scholarship and practice in mathematics education. First, in terms of curriculum theory, the paper advances inclusive curriculum discourse by conceptualizing ethnomathematics not as a peripheral enrichment activity but as a foundational curriculum principle that bridges formal school mathematics and learners' sociocultural realities. By integrating Culturally Responsive Pedagogy, Social Constructivism, and the Funds of Knowledge framework, the study offers a theoretically coherent model for understanding how culturally embedded mathematical practices can enhance relevance, equity, and epistemological access in mathematics curricula. Second, regarding Ghanaian education policy, the study provides critical insights that inform curriculum reform efforts under Ghana's Education Strategic Plan (2018-2030) and the competency-based curriculum framework by highlighting specific gaps in cultural representation, language inclusivity, teacher preparedness, and assessment practices. The findings offer evidence-based direction for policymakers, curriculum developers, and teacher education institutions on how indigenous mathematical knowledge can be systematically embedded into curriculum design, instructional materials, and professional development structures to promote inclusive education. Third, in contribution to ethnomathematics scholarship, the study extends existing literature by contextualizing ethnomathematics within a postcolonial African education system and demonstrating its relevance as a tool for curriculum transformation rather than solely classroom pedagogy. By documenting culturally situated mathematical practices such as kente weaving, Adinkra symbolism, indigenous games, and market arithmetic, the study strengthens the empirical and conceptual grounding of ethnomathematics in Ghana, thereby enriching global discussions on culturally sustaining mathematics education and knowledge democratization.

## Limitations and suggestions for further studies

Despite its theoretical depth, this study has several limitations that are directly linked to specific sections of the paper and point to clear directions for future research. First, the conceptual and literature-based nature of the study, evident in the *Introduction* and *Conceptual Framework and Theoretical Underpinnings* sections, limits the ability to empirically verify the proposed relationships between ethnomathematics, inclusivity, and learner outcomes; future studies could address this by employing classroom-based experimental or quasi-experimental designs to test whether instruction grounded in ethnomathematical practices (e.g., kente weaving or market arithmetic) leads to measurable improvements in students' conceptual understanding, engagement, and achievement. Second, while the *Ethnomathematics in the Ghanaian Context* section richly documents indigenous mathematical practices, it does not systematically assess their prevalence or instructional feasibility across regions and school levels; subsequent research could conduct regional comparative studies or large-scale surveys to examine how these cultural practices vary across ethnic and linguistic groups and how they can be aligned with specific curriculum strands. Third, the *Curriculum Analysis and Pedagogical Gaps* section identifies shortcomings in textbooks, assessments, and language use but relies on secondary analyses; future research could strengthen these claims through systematic content analysis of NaCCA-approved textbooks and assessments, using coding frameworks to quantify cultural representation. Additionally, the discussion of teacher preparedness is limited by the absence of empirical teacher data; future studies could validate the paper's claims by exploring teachers' beliefs, competencies, and instructional practices through interviews, observations, and professional development interventions. Collectively, such empirical investigations would test and substantiate the conceptual arguments advanced in this paper, transforming its theoretical propositions into evidence-based recommendations for inclusive mathematics curriculum reform in Ghana.
